# Editorial: Climate-smart livestock production: strategies for enhanced sustainability and resilience

**DOI:** 10.3389/fvets.2025.1638289

**Published:** 2025-06-27

**Authors:** Oyegunle Emmanuel Oke, Victoria Anthony Uyanga, Fisayo Oretomiloye, Monsuru Oladimeji Abioja

**Affiliations:** ^1^Department of Animal Physiology, Federal University of Agriculture, Abeokuta, Nigeria; ^2^College of Agriculture, Environmental and Human Sciences, Lincoln University, Jefferson City, MO, United States; ^3^Lethbridge Research and Development Centre, Lethbridge, AB, Canada

**Keywords:** climate change, climate-smart feeding, climate-smart management, stress, resilience, livestock sustainability

## Introduction

Livestock production systems are one of the most vulnerable sectors of the global agricultural landscape, which continues to change due to climate change (1–3). Animal health, productivity, and the overall sustainability of farming systems are increasingly at risk due to extreme weather events, prolonged heatwaves, shifting disease dynamics, and depleted natural resources (4–6). Consequently, the concept of climate-smart livestock production has received attention as an approach that promotes environmental sustainability, productivity, and adaptability to climate change. Climate-smart livestock production refers to an integrated and adaptive approach to animal production that aims to reduce greenhouse gas emissions, enhance resilience to climate variability, and sustainably increase productivity while ensuring animal welfare, food security, and environmental sustainability.

Eleven interdisciplinary contributions from 42 authors from various continents were received under the Research Topic *Climate-smart livestock production: strategies for enhanced sustainability and resilience*. These consist of reviews, conceptual viewpoints, and original research articles that discuss problems and solutions in the livestock industry. The contributions can be grouped into four sub-themes: Education and Research Trends, Policy and Financial Resilience, Technological Innovations and Management, and Nutrition and Physiology.

## Physiology and nutrition as primary adaptation techniques

One of the central themes in climate adaptation is nutrition. In order to reduce environmental effects and increase productivity, Fushai et al. highlighted climate-smart livestock nutrition in semi-arid Southern Africa. The authors advocated the strategic and sustainable use of Indigenous feed resources. Regarding physiological responses,Greene et al. investigated the effects of heat stress on the function of the ileal barrier in broilers that were divergently selected for water efficiency. They identified vulnerabilities specific to each genotype that indicate a compromise between gut integrity and water conservation. The potential of nutraceuticals in climate-resilient poultry nutrition was also highlighted by Sumanu et al., who showed the positive effects of probiotics and ascorbic acid in reducing heat stress in broilers. Additionally, Deniz et al. examined the effects of climate on equine hematology over 3 years in different species and reported a correlation between seasonal climate variation and physiological changes that are important for managing equine welfare in hot climates.

## Innovations in technology and management systems

Numerous studies highlight the contributions of systems-level management and technological innovation to achieving climate goals. Neculai-Valeanu et al. emphasized how digital technologies, like wearables and precision livestock monitoring, can improve the health and welfare of animals. The authors revealed that data could to drive the shift to precision livestock production. Furthermore, using life cycle assessment models, the findings of Thompson et al. projected that the U.S. beef and dairy industries could achieve climate neutrality by 2050 by combining interventions such as methane reduction, feed improvement, manure management, and soil carbon sequestration. In a similar vein, Sun and Wang examined carbon emissions in China's beef sector and discovered geographical differences associated with production intensity and policy. These findings are beneficial to national mitigation strategies.

## Financial and policy tools for climate resilience

Economic instruments and policy frameworks are also essential for fostering resilience. Agroforestry, integrated livestock-crop systems, and local breeding programs are among the adaptation strategies for livestock development in low- and middle-income countries (LMICs) that Bashiru and Oseni have elucidated. Extension agents and development professionals will find their recommendations helpful. Melketo et al. evaluated farmers' readiness to embrace index-based livestock insurance (IBLI) in Ethiopia (Melketo et al.). The study revealed that adoption was influenced by trust, awareness, and experience of climate shock, highlighting IBLI's potential as a risk-buffering tactic for pastoral communities.

## Trends in education, research, and future paths

Priorities for research and education are also captured in this Research Topic. In the context of climate-smart livestock management, Ritter et al. investigated different ways to improve veterinary-producer relationships. They opined that developing collaborative capacity required mutual trust, communication skills, and climate literacy. Lastly, a bibliometric analysis of worldwide research trends in livestock and climate change from 1994 to 2023 was presented by Manyike et al.. Future funding and research efforts will be guided by their findings, which have revealed significant gaps, emerging themes, and a growing scholarly output, particularly in low-income regions.

In summary, this Research Topic presents multidisciplinary, innovative approaches to climate-smart livestock production. It places a strong emphasis on systems thinking across the fields of education, policy, technology, nutrition, and genetics. The contributions demonstrate how science and innovation can promote sustainability in livestock systems, ranging from traditional knowledge to sophisticated analytics. Aligning research, policy, and practice is more crucial and needed than ever as climate variability increases. Overall, this Research Topic will stimulate workable solutions and legislative initiatives that promote climate resilience and sustainable development on a global scale.

The complete Research Topic can be accessed at https://www.frontiersin.org/research-topics/65411.
